# Probiotic Gastrointestinal Transit and Colonization After Oral Administration: A Long Journey

**DOI:** 10.3389/fcimb.2021.609722

**Published:** 2021-03-10

**Authors:** Shengyi Han, Yanmeng Lu, Jiaojiao Xie, Yiqiu Fei, Guiwen Zheng, Ziyuan Wang, Jie Liu, Longxian Lv, Zongxin Ling, Björn Berglund, Mingfei Yao, Lanjuan Li

**Affiliations:** ^1^ State Key Laboratory for Diagnosis and Treatment of Infectious Diseases, National Clinical Research Center for Infectious Diseases, Collaborative Innovation Center for Diagnosis and Treatment of Infectious Diseases, The First Affiliated Hospital, Zhejiang University School of Medicine, Hangzhou, China; ^2^ China-Canada Joint Lab of Food Nutrition and Health (Beijing), Beijing Technology & Business University (BTBU), Beijing, China; ^3^ Department of Biomedical and Clinical Sciences, Linköping University, Linköping, Sweden

**Keywords:** probiotics, colonization, adhesion, colonization resistance, gut microbiota

## Abstract

Orally administered probiotics encounter various challenges on their journey through the mouth, stomach, intestine and colon. The health benefits of probiotics are diminished mainly due to the substantial reduction of viable probiotic bacteria under the harsh conditions in the gastrointestinal tract and the colonization resistance caused by commensal bacteria. In this review, we illustrate the factors affecting probiotic viability and their mucoadhesive properties through their journey in the gastrointestinal tract, including a discussion on various mucosadhesion-related proteins on the probiotic cell surface which facilitate colonization.

## Introduction

Probiotics are defined by the FAO/WHO as “live microorganisms that, when administered in adequate amounts, confer a health benefit on the host” (Hill et al., 2014). Probiotics are gaining increasing acceptance and are now commonly used as consumer food and food supplemental products. The global market for probiotics is increasing at a compound annual growth rate of approximately 13%. Between 2010 and 2014, the global market capacity increased from US$ 25.4 billion to US$ 36.9 billion.

The effects of probiotics in disease prevention and treatment have been frequently studied. An increasing body of evidence suggests that probiotics play an active role in alleviating a variety of conditions including chronic diseases (Leung et al., 2016), infectious diseases (Shen et al., 2017), autoimmune diseases (Esmaeili et al., 2017), and pediatric diseases (Guo et al., 2019). Clinically, therapies to modulate the gut microbiota include oral administration of probiotics and fecal microbial transplantation (FMT). FMT has been proved to be an effective treatment for patients with *Clostridium difficile* infections (CDI), inflammatory bowel disease (IBD), and recurrent hepatic encephalopathy, but the applications of FMT are relatively limited compared with oral administration of probiotics (Britton and Young, 2014; Browne and Kelly, 2017; Bajaj et al., 2018). Moreover, FMT remains controversial due to the risk of the transmission of drug-resistant microorganisms which could lead to adverse infectious events (DeFilipp et al., 2019).

Compared to FMT, oral administration of probiotics has a wider range of applications and is considerably more convenient and safer. However, the viability of orally administrated probiotics is greatly challenged by harsh conditions including gastric acid, bile salts, and degrading enzymes, before they arrive at their functional site in the gastrointestinal tract (GIT) (Yao et al., 2020). Furthermore, viable probiotics reaching the colon must also manage to colonize the intestinal mucosa in competition with the indigenous bacteria (Zmora et al., 2018). Interestingly, several reports demonstrated that many of the effects obtained from viable cells of probiotics can also be realized from the dead probiotics (Adams, 2010; Li et al., 2016; Warda et al., 2019; Warda et al., 2020). Since this review mainly concentrate on the adhesion-associated surface molecules, the detailed part of dead probiotics and their function will not be described here. Although the harsh conditions in the upper GIT have been discussed in previous publications (Charteris et al., 1998; Saarela et al., 2000; Yao et al., 2020), the purpose of this review is to comprehensively illustrate the journey of probiotics from oral administration to the GIT followed by colonization of the gut, with a particular focus on the adhesion process of probiotics on the mucosa or intestinal epithelial cells.

## Transit of Probiotics Through the Gastrointestinal Tract

**Figure 1:**
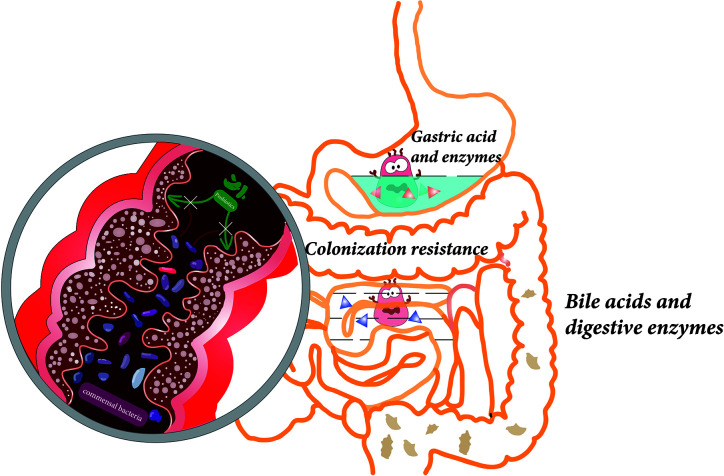
Various factors affect the viability of probiotics during gastrointestinal transit, including gastric acid, digestive enzymes, bile acids in the upper gastrointestinal tract, and colonization resistance caused by commensal bacteria in the colon.

After oral administration, probiotics pass through the GIT, from the mouth, through the stomach, to the small intestine and colon. In this section, a range of physicochemical factors (**Figure 1**), which may impact the viability of probiotics, will be described.

### Mouth

When probiotics are ingested, they will first be exposed to saliva in the mouth. Saliva is a clear and mildly acidic, mucoserous, exocrine secretion, consisting of immunologic and nonimmunologic components which protect teeth and mucosal surfaces (Humphrey and Williamson, 2001). The immunologic contents include secretory Immunoglobulin A (IgA), Immunoglobulin G (IgG), and Immunoglobulin M (IgM). The non-immunologic contents include proteins, mucins, peptides, and enzymes. Saliva has an antibacterial effect, however, it is selective and can support the growth of non-cariogenic microflora (Humphrey and Williamson, 2001). *In vitro* studies on multiple *Lactobacillus*, *Pediococcus*, and *Bifidobacteria* strains have shown no significant loss of cell count when exposed to saliva, compared with the control group (Haukioja et al., 2006; Garcia-Ruiz et al., 2014). While the transit of probiotics through the mouth and their exposure to saliva are transient after oral administration, the influence of saliva on the survival rates of probiotics seems to be minimal.

### Stomach

After passing through the esophagus, the probiotics arrive in the stomach where they are exposed to the acidic gastric fluid. The acidic environment is extremely lethal to most bacteria, especially to bacteria non-resistant to acid, which can cause a reduction of bacterial cytoplasmic pH. The influx of hydrogen ions (H^+^) leads to a decrease in activity of glycolytic enzymes, which further affects the F_1_F_0_-ATPase proton pumps. The reduction of F_1_F_0_-ATPase proton pump activity in low pH is responsible for the survival of probiotics (Yao et al., 2020). The transit through the stomach takes between 5 min and 2 h and prolonged exposure to the acidic environment is a huge challenge for the probiotics (Cook et al., 2012; Yao et al., 2020). In addition, other adverse conditions present in the stomach including ionic strength, enzyme activity (pepsin), and mechanical churning have been shown to have an impact on the viability of probiotics (Sarao and Arora, 2017; Surono et al., 2018). For example, the viable cells of *Bifidobacterium longum* and *Bifidobacterium breve* became undetectable in simulated gastric juice within an hour (Cook et al., 2012).

### Small Intestine

After passing through the pylorus, the probiotic bacteria will reach the small intestine where abundant pancreatic juice and bile are present. Under the neutralizing effect of intestinal fluid, the pH in the small intestine is about 6.0–7.0, much milder than gastric fluid (Cook et al., 2012). However, bile acids and digestive enzymes (including lipases, proteases, and amylases) can also impact probiotic viability through cell membrane disruption and DNA damage (Hamner et al., 2013; Yao et al., 2018; Yao et al., 2020). *In vitro* studies have demonstrated that the viability of *Lactobacillus salivarius* Li01 and *Pediococcus pentosaceus* Li05 is reduced in simulated intestinal fluid (Yao et al., 2017; Yao et al., 2018). To enhance the tolerance of probiotics to gastric juice and bile in the GIT, the probiotics can be coated with a protective shell, a technique known as microencapsulation. In recent years, great progress has been made in increasing the survival rate and guaranteeing that sufficient number of viable probiotics reach the colon *via* microencapsulation-based methods (Martin et al., 2015; Yao et al., 2017; Yao et al., 2018).

### Colon

The colon has the largest bacterial density (10^11^ to 10^12^ CFU/ml) where probiotics will encounter colonization resistance from commensal bacteria (O’Hara and Shanahan, 2006; Zmora et al., 2018). Probiotics must compete with the host microbiota for nutrients and adhesion sites to be able to colonize the colonic mucosa and proliferate (Zmora et al., 2018; Yao et al., 2020). Due to the colonization resistance, most probiotics are excreted out of the colon with stool after oral administration and shortly after consumption ceases so that the probiotics cannot be detected (Sierra et al., 2010; Wang et al., 2015). The mechanisms which engender the colonization resistance are illustrated in detailed in the section below.

## The Gut Microbiota and Colonization Resistance

The human body contains a huge microbiome consisting of microorganisms including bacteria, fungi, archaea, viruses, and protozoa (Shukla et al., 2017). According to previous studies, each individual contains about 10–100 trillion symbiotic microbial cells, most of which are bacteria residing in the intestines (Gilbert et al., 2018). The gut microbiota plays a symbiotic role during the development of the human body and participates in the process of maintaining health and resisting diseases (Fan and Pedersen, 2020). In this section, the composition of gut microbiota and the mechanism of colonization resistance will be discussed.

### Composition of the Gut Microbiota

The human gut microbiota consists of more than 1,000 phylotypes (Gilbert et al., 2018). In healthy individuals, most phylotypes of bacteria can be roughly classified into *Bacteroidetes*, *Firmicutes*, *Actinobacteria*, *Proteobacteria*, and *Verrucomicrobia* (Lozupone et al., 2012). Among them, *Bacteroidetes* and *Firmicutes* usually dominate the microbiota whereas *Actinobacteria*, *Proteobacteria*, and *Verrucomicrobia* are usually minor constituents. The concentration of microorganisms in the stomach and proximal small intestine is less than 10^4^ CFU/ml due to the harsh conditions in the GIT. Majorities of microorganisms inhabit in distal small intestine and colon, where the bacterial density ranges from 10^11^ to 10^12^ CFU/ml (O’Hara and Shanahan, 2006). The distribution of bacteria in the intestinal mucosa has certain ecological characteristics. Along the longitudinal axis of the intestine and colon, the oxygen concentration gradually decreases. More anaerobes such as *Clostridium* and *Faecalibacterium* reside in the lower GIT while the upper gastrointestinal tract is enriched in Gram-positive cocci (eg, *Gemella*, *Streptococcus*) (Engevik and Versalovic, 2019). Along the horizontal axis of the intestine and colon, the antimicrobial molecules and oxygen secreted from the epithelium cells accumulate at high local concentrations within the inner mucus layer, where few microbial inhabitants can colonize (Donaldson et al., 2016). The mucus layer in the colon has two different structures: a loose outer layer and a tight inner layer. The former is colonized by *Bacteroides acidifaciens*, *Bacteroides fragilis*, *Bifidobacteriaceae*, and *Akkermansia muciniphila* which can degrade mucin. The latter is penetrated at low density by a more restricted community including *Bacteroides fragilis* and *Acinetobacter* spp. (Donaldson et al., 2016).

The composition of the gut microbiota is not static. Instead, it is highly variable and its normal variation in diversity is affected by factors including age, genetics, environment, and diet (Lozupone et al., 2012; David et al., 2014; Goodrich et al., 2014; Rothschild et al., 2018). In the early years of life, especially during the first three years, the composition and function of microbes colonized in the intestine are continuously changed until a relatively stable microbial community is established. Previous studies have shown that the microbiota composition of twins and mother-daughter pairs is more similar than unrelated individuals, suggesting that genetics may play a role in the microbiota composition (Dicksved et al., 2008; Turnbaugh et al., 2009). In contrast, a recent study further showed that the microbiota composition of people living together without kinship had many significant similarities, demonstrating that host genetics had a minor role in determining microbiota composition in this case (Rothschild et al., 2018). The microbial composition is considerably different between people in different geographic locations and with different diets, indicating that the gut microbiome is significantly associated with diet and environment (Rothschild et al., 2018; Partula et al., 2019; Scepanovic et al., 2019).

### Colonization Resistance

The normal gut microbiota forms a stable bacterial community that resists the invasion of foreign bacteria and the expansion of pathogens. This phenomenon, which was discovered in 1950s, is known as “colonization resistance” (Bohnhoff et al., 1954; Freter, 1955). The mechanisms of colonization resistance can be divided into two broad categories: direct and indirect mechanisms. Among both categories, direct colonization resistance refers to restriction of exogenous microbial colonization strictly through factors associated with the gut microbiota, independently of any interaction with the host, and includes inhibition and competition for resources (Pickard et al., 2017). Indirect colonization resistance is dependent on host-derived factors, including production of antimicrobial peptides, maintenance of the epithelial barrier, and modulation of bile acid concentrations through interaction with host (Gibson et al., 2017). For example, bacteriocins are proteinaceous compounds which are synthesized in the ribosomes of both Gram-positive or Gram-negative bacteria and are able to inhibit closely related species or species that utilize similar nutrients or niches (Klaenhammer, 1993; Gibson et al., 2017). It has been found that bacteriocin-producing *Enterococcus faecalis* can inhibit the colonization of vancomycin-resistant enterococci (VRE) (Kommineni et al., 2015).

Probiotics are adversely affected by the colonization resistance exerted by the commensal gut microbiota. Some studies demonstrate that the probiotics which human beings ingest are globally shed in stool in the period confined to the time of administration and shortly thereafter (Sierra et al., 2010; Lahti et al., 2013; Wang et al., 2015). Related experiments further demonstrate that probiotics cannot change intestinal microbiota community structure or diversity (Kristensen et al., 2016; Bazanella et al., 2017; Laursen et al., 2017). Colonization resistance may be one of the important reasons for the limitation of the long-term effects of probiotics. Zmora et al. administered a combination consisting of 11 probiotic strains to adult, male specific pathogen-free (SPF) mice and germ-free (GF) mice. Stool samples were analyzed at indicated time points, followed by a dissection of the GI tract on day 28 after supplementation. Significantly higher viable counts of bacteria were observed in GF mice compared to that in SPF groups. An explanation for the results could be that the probiotics encounter a higher degree of mucosal colonization resistance in the SPF mice compared to in the GF mice (Zmora et al., 2018). Another interesting study indicated that the efficacy of probiotic colonization varies among different persons. Volunteers were divided into two groups, “permissive” and “resistant.” People in the permissive group had a significant increase in probiotic strains in their intestinal mucus membrane, whereas probiotics were not detected in the intestine of people in the “resistant” (Zmora et al., 2018).

## Probiotic Colonization of the Intestinal Mucosa

Successful colonization of the gastrointestinal tract is a key factor for probiotics to be able to exert a sufficient host-interaction to confer health benefits (Alp and Kuleasan, 2019). Mucosal adhesion is considered a critical step in probiotic colonization; however, the mechanisms of adhesion still require exploring. In this section, we discuss the composition of the intestinal mucus layer and specific proteins related to probiotic adhesion.

### Intestinal Mucosa and Mucus Layer

The intestinal mucosa is composed of epithelial layer, lamina propria, and muscularis mucosa. Small intestinal villi, which are formed by the epithelium and lamina propria protruding into the intestinal cavity, cover the surface of the mucosa and are responsible for the absorption of nutrients in the intestine. The epithelial cells are composed of absorptive cells, goblet cells and endocrine cells. Goblet cells are scattered between absorptive cells, secreting mucus which covers the entire small intestinal cavity, composed of carbohydrates, lipids, salts, protein, bacteria, and cellular debris (Ensign et al., 2012). The thickness of mucus varies from approximately 30 to 300 μm; the thickness increases from the intestine to the rectum (Van Tassell and Miller, 2011). The main proteins are glycoproteins called mucins which polymerize to form a continuous gel matrix, providing a structural basis for the mucosal layer, protecting the intestine from pathogens, enzymes, toxins, dehydration, and abrasion. At the same time, exogenous nutrients such as vitamins and minerals are present in the intestinal mucus, which provide a huge ecologic growth advantage for bacteria colonized in the intestinal mucus (Sicard et al., 2017). It can be said that the mucus is an excellent niche for both of probiotics and pathogen.

### Adhesion

The process of bacterial adhesion to the mucosa includes reversible and stable stages (Kos et al., 2003). Initially, probiotics bind to the mucosa through non-specific physical contact, including spatial and hydrophobic recognition, establishing reversible and weak, physical binding (Van Tassell and Miller, 2011). Subsequently, with the specific interactions between adhesins (usually proteins anchored on the cell surface) and complementary receptors, probiotics establish a stable binding to the mucus or intestinal epithelial cells (IECs), thereby successfully colonizing the GIT (Van Tassell and Miller, 2011).

**Figure 2:**
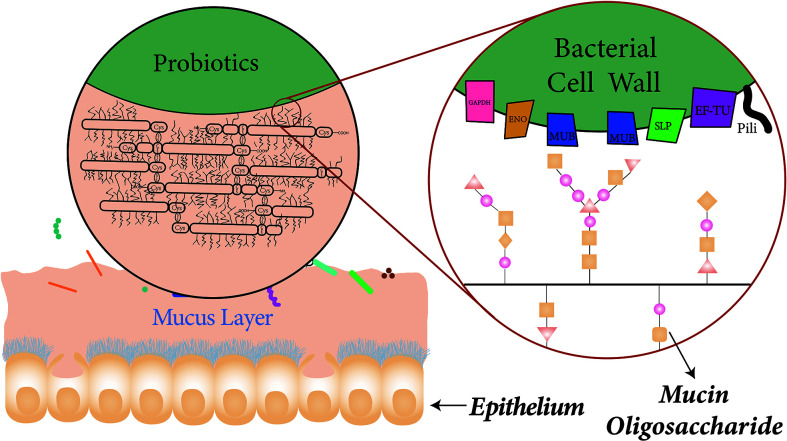
The composition of the mucus layer and association with probiotic surface proteins. Goblet cells are scattered between absorptive cells, which can secret mucus that cover the entire small intestinal cavity. The mucus is mainly composed of mucins which are rich in cysteine. The extensive disulfide bonds between mucins form the characteristic viscoelastic properties of mucus. The specific proteins on the surface of probiotics play an important role in probiotic adhesion to mucus. Mucus-binding proteins for example, can bind to the mucus layer through interactions with glycosyl modifications of mucin.

Probiotics can encode numerous cell-surface factors which are involved in adherence to mucin or IECs. Buck et al. inactivated and knocked out several specific cell surface factors in the *Lactobacillus acidophilus* NCFM, including mucin-binding protein (Mub), fibronectin-binding protein (FbpA), and surface layer protein (SlpA). Significant decrease in adhesion to Caco-2 cells was observed in the each separate protein mutant, showing that the genes which encode FbpA, Mub, and SlpA all contribute to *L. acidophilus* NCFM adhesion to IECs *in vitro* (Buck et al., 2005). Another similar *in vitro* study found that mutations in *luxS* in *L. acidophilus* NCFM, which encodes autoinducer (AI)-2, caused a decrease in the adhesion to IECs (Buck et al., 2009). Additional work demonstrated the involvement of myosin cross-reactive antigen (MCRA) of *L. acidophilus* NCFM in adhesion to Caco-2 cells (O’Flaherty and Klaenhammer, 2010) and the deletion of the gene encoding sortase from *L. salivarius* resulted a significant reduction in adhesion to human epithelial cell lines (van Pijkeren et al., 2006). In addition to the proteins, there are also non-protein molecules present in probiotics, including teichoic acids (TA) and exopolysaccharides (EPS) which can interact with host cells to influence the adhesion. It can be inferred from current publications that there is no fixed molecule that can be applied to all strains of probiotics, despite of the wide range of adhesion-related molecules. Many adhesins seem to be specie or strain dependent. These adhesion-associated surface molecules of probiotics and mechanisms related to adhesion are discussed in detail below (**Table 1** and **Figure 2**).

**Table 1:** Adhesion-related molecules in probiotics.

Proteins	Adhesion-related function	Probiotics	References
**MUBs**	Binds to mucus *in vitro*	*L. reuteri*	(Roos and Jonsson, 2002; MacKenzie et al., 2009; Jensen et al., 2014)
**FnBPs**	Binds to fibronectin	*L. acidophilus* *L. casei* *Bacillus subtilis*	(Schillinger et al., 2005) (Munoz-Provencio et al., 2010) (Rodriguez Ayala et al., 2017)
**SLPs**	Expression levels of SLP are related to the adhesion capability	*L. acidophilus* *P. freudenreichii*	(Buck et al., 2005) (do Carmo et al., 2017)
**SLPAs**	Binds to mucins and IECs	*L. acidophilus* *L. helveticus*	(Hymes et al., 2016; Klotz et al., 2020) (Johnson and Klaenhammer, 2016)
**ENO**	Binds to ECM, null mutants display diminished adhesion	*L. plantarum* *B. bifidum*	(Castaldo et al., 2009) (Wei et al., 2016)
**GAPDH**	Binds to human colonic mucin	*L. plantarum* *L. acidophilus*	(Kinoshita et al., 2008) (Patel et al., 2016)
**EF-TU**	Binds to Caco-2 cells and mucin	*L. plantarum* *L. johnsonii* *L. paracasei a/L. casei* *B. longum*	(Ramiah et al., 2008) (Granato et al., 2004) (Zhang et al., 2016) (Nishiyama et al., 2020)
**GroEL**	Binds to mucins and IECs	*L. johnsonii* *B. longum*	(Bergonzelli et al., 2006) (Nishiyama et al., 2020)
**APF**	Binds to mucins and epithelial cells	*L. acidophilus* *L. gasseri*	(Goh and Klaenhammer, 2010) (Nishiyama et al., 2015)
**Pili**	Play a role in the adhesion to ECM and IECs	*L. rhamnosus* *L. lactis* *B. bifidum, B. breve, B. longum, and B. adolescentis*	(Kankainen et al., 2009; Lebeer et al., 2012; Rintahaka et al., 2014) (Meyrand et al., 2013) (Westermann et al., 2016)
**EPS**	Play a role in the interaction with host cells	*L. plantarum* *L. rhamnosus GG* *L. johnsonii* *L. reuteri* *B. animalis* *B. longum*	(Lee et al., 2016) (Lebeer et al., 2009) (Dertli et al., 2015) (Sims et al., 2011) (Castro-Bravo et al., 2017) (Tahoun et al., 2017)
**TA**	Inhibit adhesion to Caco-2 cells	*L. johnsonii*	(Granato et al., 1999)

#### Mucus-Binding Proteins

Mucus-binding proteins (MUBs) are cell surface proteins with a typical signal peptide and C-terminal LPxTG motif in the C-terminus which establish a covalent binding to the bacterial cell wall (Juge, 2012). MUBs are usually found in lactic acid bacteria, especially *Lactobacillus reuteri*, which is one of the most dominant probiotic bacteria in the human GIT (Roos and Jonsson, 2002; MacKenzie et al., 2009; Jensen et al., 2014). MUBs contain multiple Mub repeats (Mub domains, ~200 residues) which share homology to the mucin-binding protein repeats (MucBP domains, ~50 residues) (Mercier-Bonin and Chapot-Chartier, 2017). Mub domains can be found in proteins of numerous *Lactobacillus* spp., including *L. acidophilus*, *L. plantarum*, *L. brevis*, and *L. fermentum* (Van Tassell and Miller, 2011). The amino acid sequence of Mub is highly repetitive and contains two types of related repeats, Mub1 and Mub2. Single antibodies against Mub1 and Mub2 ​​had no inhibition on adhesion experiments, demonstrating that the repetitive structure of both is important for the progress of adhesion (Roos and Jonsson, 2002). Experiments have also suggested that Mub interacts with carbohydrate components on the mucus, particularly with the glycosylic bond of mucins (Van Tassell and Miller, 2011). The distribution of MucBP domains in bacterial proteins is more extensive than that of Mub (Juge, 2012). Similarly, MucBPs in *Lactobacillus* have been demonstrated to be able to bind to mucus (Radziwill-Bienkowska et al., 2016).

#### Fibronectin-Binding Proteins

The extracellular matrix is a complex network of large molecules outside the cells in which the extracellular glycoprotein fibronectin is ubiquitously present. Fibronectin-binding proteins, which are anchored on the bacterial surface, belong to the microbial surface components recognizing adhesive matrix molecules (MSCRAMM) family of adhesins (Schwarz-Linek et al., 2006). It has been shown that fibronectin-binding proteins present on the surface of *L. acidophilus* can bind to the exposed fibronectin and anchor the IECs (Schillinger et al., 2005). Munoz-Provencio et al. showed that purified fibronectin-binding protein, encoded by *fbpA* of *Lactobacillus casei* BL23, could bind to immobilized fibronectin. They also observed that mutants with inactivated *fbpA* showed a lower adhesion rate to immobilized fibronectin (Munoz-Provencio et al., 2010).

#### Surface-Layers Proteins

The outermost strata of the bacterial cell wall consist of the surface (S-) layers, non-covalently bonded semi-porous crystal arrays comprised of self-assembling proteinaceous subunits called S-layer proteins (SLPs) (Sara and Sleytr, 2000). The lattices of the S-layer exhibit oblique, square, or hexagonal symmetry when observed with an electron microscope. Most S-layers are 5 to 25 nm thick and have a molecular weight of almost 40–200 kDa. S-layers have been found in hundreds of species in almost every taxonomic group of walled bacteria (Sleytr et al., 2014). S-layers have been shown to be involved in a number of processes including maintaining cell shape, protecting the murein sacculus from lysozyme attack, acting as molecular sieves and antifouling coating, serving as binding sites, and promoting bacterial adhesion (Sleytr et al., 2014). SLPs of probiotics also have many benefits to the host. Recent studies found that SLPs purified from *Lactobacillus* exerted immunomodulatory effects, which attenuated intestinal barrier dysfunction and inflammation, and protected intestinal epithelial barrier (Prado Acosta et al., 2016; Zhang et al., 2017; Wang et al., 2019).

Surface-layer protein A (SlpA) is a S-layer protein specifically found in *L. acidophilus* NCFM. Knockout of SlpA engendered decreased adhesive capability of the bacteria (Buck et al., 2005). Ashida et al. compared adhesive capabilities of eight *L. acidophilus* strains to Caco-2 cells and found that the adhesive capability of *L. acidophilus* L-92 was highest and that of *L. acidophilus* CP23 was lowest among the compared strains (Ashida et al., 2011). Further research showed that the expression levels of SlpA on the surface of *L. acidophilus* L-92 was about 40-fold higher than that of *L. acidophilus* CP23 (Ashida et al., 2011). In *Propionibacterium freudenreichii* CIRM-BIA 129, another protein called surface-layer protein B (SlpB), have also been shown to play a key role in adhesion to human intestinal cells. Significant inhibition of adhesion to HT-29 cells was observed when blocking SlpB with specific antibodies or when inactivating *slpB* in *P. freudenreichii* CB129 (do Carmo et al., 2017).

Johnson et al. identified proteins covalently, co-localized to the outermost stratum of the cell surface within the S-layer of *L. acidophilus* NCFM, designated as S-layer associated proteins (SLAPs) (Johnson et al., 2013). SLAPs have subsequently been characterized in several *Lactobacillus* spp. (*L. helveticus*, *L. crispatus*, *L. amylovorus*, and *L. gallinarum*) (Johnson et al., 2016). Both SLPs and SLAPs are important mediators of adhesion to host IECs and mucins (Buck et al., 2005; Hymes et al., 2016; Johnson and Klaenhammer, 2016; Klotz et al., 2020). Interestingly, one of the most prevalent SLAPs in *L. acidophilus* NCFM, PrtX, acts as a serine protease homolog, and has been shown to be negatively correlated with adhesion in *in vitro* experiments (Johnson et al., 2017). In the study by Johnson et al. the gene *prtX*, was deleted from the chromosome of *L. acidophilus* NCFM and it was discovered that the PrtX-deficient strain (Δ*prtX*) showed an enhanced cell binding ability to mucin and fibronectin compared to the wild type strain (Johnson et al., 2017). More effects of SLPs and SLAPs on the adhesion are still waiting for exploring.

#### Moonlighting Proteins

Moonlighting proteins are defined as multifunctional proteins which can exhibit more than one biological function (Jeffery, 1999). Almost 400 moonlighting proteins have been discovered which can be found at MoonProt Database (http://www.moonlightingproteins.org). Moonlighting proteins including enolase (ENO), glyceraldehyde-3-phosphate dehydrogenase (GAPDH), elongation factor-Tu (EF-Tu), and molecular chaperones have been demonstrated to be involved in adhesion of probiotics to human intestinal mucins or IECs (Bergonzelli et al., 2006; Siciliano and Mazzeo, 2012). A more detailed description of the involvement of specific moonlighting proteins in adhesion follows below.

##### Enolase

Enolase is a multifunctional protein which plays a key role in variety of pathophysiological processes such as glycolysis, fibrinolysis, and DNA transcription (Pancholi, 2001). As a moonlighting protein, enolase was discovered on the *L. plantarum* LM3 and *B. bifidum* S17 cell surface and it was shown that the protein could bind specifically to the extracellular matrix, thus facilitating the adhesion of bacterial cells to the host (Castaldo et al., 2009; Wei et al., 2016). Castaldo et al. also compared the differences between wild type strains and mutant strain which carried the enolase null mutation and showed the adhesion ability of mutant strain was less efficient than that of wild strain (Castaldo et al., 2009).

##### Glyceraldehyde-3-Phosphate Dehydrogenase

Glyceraldehyde-3-phosphate dehydrogenase (GAPDH) is an enzyme involved in the glycolysis. GAPDH is considered as a moonlighting protein because it has diverse functions in different processes, including in regulation of apoptosis (Hara et al., 2005), iron homeostasis (Rawat et al., 2012), and transcription activation (Zheng et al., 2003). GAPDH catalyzes enzymatic reactions mainly in the cytosol. Moreover, it has also been indicated that GAPDH is able to bind the cytoskeletal and extracellular matrix proteins on the cell surface of group B streptococci (Seifert et al., 2003). GAPDH lacks an extra-cytoplasmic sorting sequence, and it is interesting how the GAPDH transfers from cytosol to the cell surface (Siciliano and Mazzeo, 2012). One study showed that *L. plantarum* LA 318 adheres to human colonic mucin by GAPDH which is expressed on the cell surface (Kinoshita et al., 2008). Similarly, Patel et al. (2016) cloned the gene encoding GADPH from *L. acidophilus*, and expressed, purified, and obtained a recombinant product (r-LaGAPDH). It was discovered that the recombinant protein was in tetramer form in solution, and it showed mucin binding and hemagglutination activity. Several studies have found that in addition to binding to mucin, GAPDH of *L. plantarum* also has a highly specific adhesive capacity to plasminogen and fibronectin (Sanchez et al., 2009; Glenting et al., 2013).

The stress response of probiotics when exposed to gastric juice and bile will have an effect on the adhesive capacity to mucins and IECs. Agustina et al. reported that the adhesion of *L. paracasei* strains to mucin and IECs increased after gastrointestinal acid and bile stress. It is demonstrated that the increased adhesive capacity was attributed to the positive modification of GAPDH biosynthesis (Agustina Bengoa et al., 2018). However, bile or acid stress does not always result in increased adhesion capacity. For example, *L. delbrueckii* subsp. *lactis 200* and *L. delbrueckii* subsp. *lactis 200+* grown in medium containing bile showed a decrease in adhesion to IECs (Burns et al., 2010).

##### Elongation Factor Tu

Elongation factor Tu (EF-Tu) is an intracellular protein which serves several functions in protein synthesis and protein folding, including facilitating protein synthesis and increasing translation accuracy (Beck et al., 1978). EF-Tu is comprised of three domains known as domains I, II, and III, forming different sites for binding of guanosine triphosphate (GTP) and aminoacyl-tRNA (Harvey et al., 2019). This structure enables EF-Tu to transport aminoacyl-tRNAs to the ribosome during protein synthesis. Interestingly, EF-Tu is a highly conserved protein which can be found on both cell surfaces of pathogens and probiotics (Kunert et al., 2007; Espino et al., 2015; Thofte et al., 2018). The role of EF-Tu on the cell surface involves the processes of bacterial adhesion to host cells, invasion, and immune evasion (Ramiah et al., 2008; Lopez-Ochoa et al., 2017). Zhang et al. used 5 M LiCl to remove the surface proteins (EF-TU and surface antigen) of *L. paracasei* and *L. casei*. After treatment, their adhesion force to HT-29 cells significantly reduced (Zhang et al., 2016). Nishiyama et al. found that *B. longum* can release particles into the extracellular environment and relevant proteomics analysis identified several mucin-binding proteins, including EF-Tu (Nishiyama et al., 2020).

##### Molecular Chaperones

Molecular chaperones are a large class of proteins which facilitate binding and stabilization of unstable conformations of other proteins, and promote correct folding of intracellular proteins (Ellis, 1987). GroEL is a molecular chaperone which assists the folding of nascent or stress-denatured polypeptides through binding and encapsulation (Clare et al., 2012), and has additionally showed moonlighting functionality, including binding activity to mucins and IECs (Bergonzelli et al., 2006). It has also been indicated in *in vitro* studies that GroEL plays a critical role in the binding process of *L. johnsonii* La1 to mucus and intestinal cells in the host environment. Interestingly, the binding process of GroEL to mucins or intestinal cell lines was pH-dependent and the binding capacity varied with the pH; the binding capacity was higher at pH 5.0 compared to that at pH 7.2 (Bergonzelli et al., 2006). Small heat shock proteins as ATP-independent chaperones (sHsps) act by binding unfolding proteins, thereby delaying the formation of harmful protein aggregates (Janowska et al., 2019). sHSPs contribute to cellular defense against harsh conditions under physiological conditions and the GIT stress responses of most bacteria involving the upregulation of sHSPs (Guzzo, 2012; Haslbeck and Vierling, 2015; Khaskheli et al., 2015). Nishiyama et al. compared the adhesion ability of 31 *L. pentosus* strains to mucin and discovered a highly adhesive *L. pentosus* strain, which over-produced four moonlighting proteins including sHSPs (Pérez Montoro et al., 2018). A recent study investigated the impact of knockout of the sHSP genes (including HSP1, HSP2, and HSP3) on adhesion of *L. plantarum* WCFS1 to human enterocyte-like cells, demonstrating that sHSP genes deletion lowered GIT stress resistance and adhesion capacity (Longo et al., 2020).

#### Aggregation-Promoting Factors

Aggregation-promoting factors (Apf) are secreted proteins which induces self-aggregation and facilitates the maintaining of cell shape. These proteins have mainly been found among *Lactobacillus* spp. (Nishiyama et al., 2015). It has been found that Apf-deficient mutants of *L. acidophilus* NCFM showed a significant reduction of adherence to Caco-2 cells and mucins compared with the wild type strain, suggesting Apf acts as an adhesion factor which participates in the interaction with the host mucus layer and IECs (Goh and Klaenhammer, 2010). Similar results have been shown in *L. gasseri* SBT2055 (Nishiyama et al., 2015).

#### Pili

Pili are short, straight, and filamentous structures stretching from the cell surface of bacteria. Pili are mostly characterized among Gram-negative bacteria. However, pili-like structures are also found in probiotics like *Bifidobacterium* spp. and *Lactobacillus* spp. (Alp and Kuleasan, 2019). Unlike those in Gram-negative bacteria, these pili have a narrow diameter (~1–10 nm) and every pilus consists of multiple pilin subunits which are coupled to each other covalently (Kankainen et al., 2009). Lankainen et al. discovered three LPXTG-like pilins (SpaCBA) in *L. rhamnosus GG* (LGG) (Kankainen et al., 2009). Each of the three pilins has its own location and function in the pilus: backbone SpaA for length, basal SpaB for anchoring, and tip SpaC for adhesion (Kant et al., 2020). Study showed the adhesion to human intestinal mucus was destroyed by SpaC antibody and blocked in a mutant of LGG which carried the inactivated SpaC gene, demonstrating the SpaC is essential in the interaction with mucus (Kankainen et al., 2009; Lebeer et al., 2012). Subsequently, another type of LGG pilus called SpaFED was phenotypically characterized. Similar to SpaCBA, SpaFED pilus can also mediate the adhesion to mucin (Rintahaka et al., 2014). Meyrand et al. detected one adhesion-associated pilin on the surface of *L. lactis* which was plasmid-encoded, suggesting the possibility of spread of adhesion effect among *L. lactis* through horizontal gene transfer (Meyrand et al., 2013). Type Via pili, type IVb tight adherence (Tad) pili, and sortase-dependent pili have been found in the genomes of almost *Bifidobacterium* spp., including *B. bifidum*, *B. breve*, *B. longum*, and *B. adolescentis*, and have been demonstrated to play important roles in the adhesion to IECs or the extracellular matrix (Westermann et al., 2016). A recent study showed that acid stress could enhance the adhesion ability of GG to intestine epithelium through the induction of pili-related genes including spaC and spaF (Bang et al., 2018).

#### Exopolysaccharides

Exopolysaccharides (EPS) are surface carbohydrate polymers existing in most bacteria and fungi. They have various bioactivities functions, including lowering cholesterol, immunomodulating, anti-oxidation, anti-virus, counteract colonization of enteropathogens, and anti-coagulant (Fanning et al., 2012; Zivkovic et al., 2015; Zhou et al., 2019). As a protective surface layer, EPS play a positive role in helping probiotics enhance the tolerance to harsh condition of GIT by forming biofilms and communicating with other microorganisms or with host cells (Arena et al., 2017). However, there has been no conclusive conclusions so far about whether EPS can promote adhesion. According to existing references, EPS can not only participate in the adhesion process, but also reduce the adhesion efficiency of probiotics. Since the EPS on the probiotic surface, especially those with high molar mass and large volume, may shield other adhesion proteins. One previous report estimated the adhesive properties of several lactic acid bacteria (LAB) strains to Caco-2 cells, and found EPS may facilitate probiotic adhesion (Garcia-Ruiz et al., 2014). The effect of EPS on bacterial adhesion seems to be dependent on probiotic specie and strain. A previous study investigated three EPS depletion mutant strains of *L. plantarum*. Lp90 mutant strain showed improved adhesion to Caco-2 cells compared to the Lp90 wild-type strain. Interestingly, the depletion of EPS genes for WCFS1 and SF2A35B strains did not influence their mucoadhesion (Lee et al., 2016). For *B. animalis*, higher proportion of high molecular weight of EPS showed lower mucoadhesion, indicating that different EPS on bacterial surface might confer variable adhesion characteristics (Castro-Bravo et al., 2017). Although the contribution of EPS to the probiotic colonization process is controversial, it can be confirmed that the presence of EPS plays a significant role in the interaction of probiotics with the host.

#### Teichoic Acids

Teichoic acids (TAs) are important components of the Gram-positive bacterial cell wall, which are composed of alditol phosphate repeating units, contributing to the hydrophobic character and electrostatic charge of the bacterial cell surface (Arena et al., 2017; Wu et al., 2020). TA can be divided into lipotheicoic acid (LTA) and wall teichoic acid (WTA). In early 1980s, the role of both TA on binding to host cells was raised (Beachey, 1975; Aly et al., 1980). One study found that LTA could inhibit the adhesion of *L. johnsonii La1* to Caco-2 cells in a concentration-dependent way (Granato et al., 1999).

## Conclusions

We discussed various unfavorable conditions which influence the viability and mucoadhesion of probiotics during GI transit. Colonization of probiotics on the mucus layer could be achieved when adhesive proteins from each side bind together, on the premise of overcoming the colonization resistance. Thus, the characteristics and functions of different proteins of were specifically reviewed. However, most of current research on mucoadhesion-related molecules of probiotics are limited to lactic acid bacteria. Adhesive proteins and mucoadhesion mechanisms of probiotics such as Bifidobacterium, Enterococcus, Pediococcus are still waiting for exploring. Besides, how probiotics communicate with commensal bacteria and some are successfully introduced to gut microbiota is also of great interest. Understanding these factors will facilitate the employment of effective delivery strategies designed for probiotics to overcome colonization resistance and achieve health benefits.

## Author Contributions

SH developed the idea of the manuscript, drafted the manuscript, and edited the manuscript. YL, JX, and YF helped with the figures and the table. BB, ZL, and LXL revised the manuscript. ZW and JL developed the idea of the manuscript, drafted the outline, and revised the manuscript. MY and LJL organized and edited the manuscript. All authors contributed to the article and approved the submitted version.

## Funding

This work was supported by the National Key Research and Development Program of China (2018YFC2000500) and National Natural Science Foundation of China (32001683).

## Conflict of Interest

The authors declare that the research was conducted in the absence of any commercial or financial relationships that could be construed as a potential conflict of interest.
